# Plant Food Allergens: Another Climate Change–Public Health Link

**DOI:** 10.1289/ehp.0900670

**Published:** 2009-05

**Authors:** Paul John Beggs

**Affiliations:** Environmental Science, Department of Environment and Geography, Faculty of Science, Macquarie University, New South Wales, Australia, E-mail: paul.beggs@mq.edu.au

The recent article titled “Rising CO_2_, Climate Change, and Public Health: Exploring the Links to Plant Biology” (Ziska et al. 2009) is an interesting and useful commentary on this important topic. Although some aspects of the article have been considered in some detail previously, such as the impacts of climate change and elevated carbon dioxide on aerobiology (e.g., Beggs 2004; Confalonieri et al. 2007) and the human health implications of this (e.g., Beggs and Bambrick 2005; Shea et al. 2008), the broader review of links between climate change, plant biology, and human health, particularly the examination of toxicology and pharmacology, is timely and brings together a number of somewhat distinct areas of research.

In their article, Ziska et al. (2009) mentioned the potential impacts of elevated atmospheric CO_2_ concentration on ragweed pollen allergenicity and poison ivy toxicity, and therefore considered respiratory allergies and contact dermatitis, respectively. Surprisingly, however, these authors did not recognize that there is a third major mechanism for human contact with plant allergens: ingestion. In an article published in *Air Quality, Atmosphere & Health*, I (Beggs and Walczyk 2008) proposed, for the first time, a link between climate change and plant food allergens, such as peanut, and the rise of associated diseases. There is potential for such impacts in the future, but they may have already occurred (Beggs and Walczyk 2008).

I agree that the plant biology aspect of climate change and human health is underappreciated, and I also strongly support Ziska et al.’s (2009) highlighting of the many key questions that remain to be addressed and the urgent need to find answers to these questions. Studies should also investigate the impacts of climate change, particularly elevated CO_2_, on plant food allergens, especially their relative concentrations.

## Editor’s note

In accordance with journal policy, Ziska et al. were asked whether they wanted to respond to this letter, but they chose not to do so.
